# Inadequate evaluation and management of suspected ­infections after TKA surgery in Lithuania: a retrospective study of 2,769 patients with 2-year follow-up

**DOI:** 10.1080/17453674.2019.1614763

**Published:** 2019-05-09

**Authors:** Egle Terteliene, Kazimieras Grigaitis, Otto Robertsson, Justinas Stucinskas, Sarunas Tarasevicius, Narunas Porvaneckas, Algirdas Venalis

**Affiliations:** a Vilnius University, Faculty of Medicine, Vilnius, Lithuania;; b Department of Orthopedics, Medical Academy, Lithuanian University of Health Sciences, Kaunas, Lithuania;; c Department of Clinical Sciences and Department of Orthopedics, Lund University and Lund University Hospital, Lund, Sweden;; d State Research Institute Center for Innovative Medicine, Vilnius, Lithuania

## Abstract

Background and purpose — The evidence-based algorithms for treatment of periprosthetic joint infection (PJI) recommend surgical intervention in combination with the use of systemic antibiotics. However, still it is not unusual to treat total knee arthroplasty (TKA) patients with suspected infection using only antibiotics. We investigated treatment pathways for TKA patients with suspected infection in Lithuania.

Patients and methods — Of the 4,069 TKA patients (4,269 knees) registered in the Lithuanian Arthroplasty Register (2013–2015) 2,769 patients (2,825 knees) were interviewed 2 years after the surgery. The patients were asked if they had been subject to antibiotic treatment after the TKA surgery and/or if any additional surgical interventions on the operated knee had been performed. The number of patients treated with antibiotics due to problems in the operated knee was identified and cumulative revision rates (CRR) were calculated.

Results — 180 (7%) patients of the total 2,769 reported that they had been prescribed antibiotics after the primary TKA; 132 of these patients (70%) said they had received antibiotics due to problems with the operated knee. The 2-year CRR after TKA in patients not treated with antibiotics was 0.7% (95% CI 0.4–1), as compared with 24% (95% CI 17–32) in those who had used antibiotics due to the problems in the operated knee for more than 1 week.

Interpretation — In Lithuania there seems to be a lack of adherence to evidence-based treatment guidelines when infection is suspected after primary TKA.

Periprosthetic joint infection (PJI) after total knee arthroplasty (TKA) is recognized as the most frequent reason for revisions, especially in the early postoperative stage (Kurtz et al. 2010). Most studies report a 1–2% incidence of PJI about after primary TKA (Peersman et al. 2001, Phillips et al. 2006, Kurtz et al. 2010, Matsen Ko et al. 2016). Accurate and early diagnosis of postoperative PJI and adequate treatment is the key to success. Currently, the evidence-based algorithms concerning the diagnosis and treatment of periprosthetic joint infections of the hip and knee indicate that only surgical treatment such as a debridement, antibiotics, irrigation, and retention of the prosthesis (DAIR) procedure or a 1- or 2-stage revision combined with systemic antibiotic treatment is to be recommended (Azzam et al. 2010, Parvizi et al. 2010, Osmon et al. 2013, Ghanem et al. 2014, Frank et al. 2017, Grammatopoulos et al. 2017). However, in “real life” some patients are still prescribed antibiotics without having surgical intervention in the hope that redness, tenderness, or wound leakage is not a serious infection and that surgical intervention can be avoided (Wagenaar et al. 2017). However, such usage of antibiotics may lead to increased bacterial resistance and more complicated treatment of an infected prosthesis, where matured biofilm on the prosthetic surface can no longer be eradicated with antibiotics only (Bjarnsholt et al. 2013). We evaluated how suspected infection after TKA was treated in “real life” in Lithuania with respect to adherence to guidelines, and investigated the outcome of antibiotic treatment without surgical intervention.

## Patients and methods

Data on patients having primary TKA procedures was derived from the Lithuanian Arthroplasty Register (LAR) (Tarasevicius et al. 2014) in order to be able to contact operated patients with an inquiry regarding their use of antibiotics during the first 2 years after the primary procedure. The completeness in the LAR was investigated in 2016, by comparing the register with State Patients fund data, and was 95% for primary TKA and 98% for revisions.

**Figure 1.**
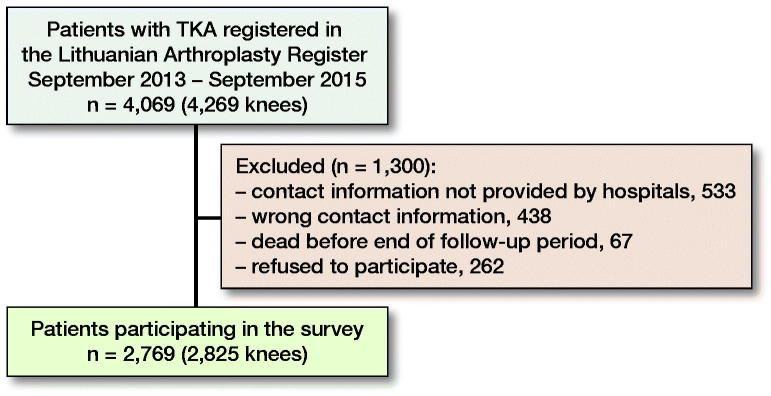
Description of material and patients interviewed regarding their use of antibiotics after surgery.

4,269 primary TKAs operated in 22 hospitals were registered in LAR between September 1, 2013 and September 1, 2015. 2,825 TKAs (2,769 patients) were included in the study (Figure 1).

The patients were approached by 1 of the researchers at 2 years after the primary TKA. The following questions were asked: Have you used an antibiotic after your primary TKA? When did you start using antibiotics? For how long did you use antibiotics? What was the reason for the antibiotic’s usage? Who prescribed the antibiotics? Patients who responded as having used antibiotics for problems in the operated knee were additionally asked if they had been the subject of puncture. Finally, we asked whether the respondents had undergone revision at any time during the 2 years after the primary TKA. After the interview the hospital that had performed the procedure was asked to provide the relevant medical charts to ascertain that the additional surgery performed was a true revision according to the LAR definition. Revision in the LAR was defined as addition, exchange, or removal of 1 or all components.

The patients were divided into 3 groups. Group 1 comprised those who received antibiotic treatment because of problems with their knee for a period of more than 1 week during the first 2 years after the primary TKA. Group 2 included those who received antibiotic treatment for more than 1 week due to problems not related to the operated knee and Group 3 patients were those having not had antibiotic treatment or who had treatment for 7 days or less.

### Statistics

For descriptive statistics, we used frequencies and ranges. Statistical evaluation included 95% confidence intervals (CI). The cumulative revision rate (CRR) was calculated with Kaplan–Meyer statistics and graphs plotted with CI for all groups; a p-value < 0.05 was considered significant. STATA v13 (StataCorp 2013) was used for calculations.

### Ethics, funding, and potential conflicts of interests

The study was approved by the national ethical committee (No. 158200-16-832-371, approved on 2016-06-15). No funding were received to conduct the study and no conflict of interests needs to be declared.

## Results

188 (7%) of 2,769 patients responded “yes” to the question: “Have you used antibiotics after the primary TKA?” When asked for the reason why antibiotics had been prescribed, 132 (Group 1) of the 188 patients (70%) said they had received antibiotics due to problems with the operated knee, while 56 (Group 2) (30%) had received the antibiotics for reasons other than the operated knee (pneumonia, bronchitis, urinary tract infection, tonsillitis). Of the 132 patients (Group 1), 68 (52%) reported that the reason for the antibiotic treatment had been infection prophylaxis, while the remaining 64 patients (49%) reported that the reason for the treatment had been that the physician had suspected a prosthetic joint infection (redness, pain, swelling of the operated knee, wound leakage). Patients receiving antibiotic treatment either for prophylaxis or due to suspected infection did not differ significantly from non-antibiotic users’ TKA with regard to their age, sex, and preoperative diagnosis. Among those 132 TKA patients who were prescribed antibiotics because of knee problems the prescribing physician was an orthopedic surgeon in 96 cases (73%) and 34 (26%) reported having used antibiotics for more than 1 month. Of the patients in Group 1, 32 reported that they had had a knee aspiration. Of these, 23 were subsequently revised, 21 because of infection. 100 of the patients in group 1 were not aspirated (Table 1).

**Table 1.** Outcome of survey in TKA patients who received antibiotics due to problems with the operated knee (Group 1). Values are frequency (%)

Received antibiotics due to problems with the operated knee (Group 1)	Patients (%)
Number of TKA patients/total no. of patients	132/2,769 (4.8)
As prophylaxis	68/132 (52)
As treatment	64/132 (48)
Antibiotics prescribed by orthopedic surgeon	96/132 (73)
Antibiotics for more than 1 month	34/132 (26)
Diagnostic knee aspiration performed	32/132 (24)

Among the 132 patients who had antibiotic treatment (Group 1), 32 had been subject to revision surgery within 2 years of the primary operation. None had been revised in Group 2 (using antibiotics for other reasons) and 23 patients among the 2,581 (Group 3) who reported no antibiotic usage had undergone revision.

The reason for revision was infection in 22 patients in Group 1 and in 3 among the non-antibiotic users (Group 3) (Table 2).

**Table 2.** Reasons for revision in Groups 1, 2, and 3. Values are frequency (%)

Revision diagnosis	Group 1 n = 132	Group 2 n = 56	Group 3 n = 2,581
Infection	22 (17)	0	3 (0.1)
Loosening of tibial component	5 (3.8)	0	0
Dislocation of the patella	0	0	1 (0.04)
Pain in the patella	0	0	5 (0.2)
Pain for unknown reason	1 (0.8)	0	2 (0.08)
Limited range of motion	0	0	3 (0.1)
Loosening of femoral component	1 (0.8)	0	2 (0.08)
Instability	1 (0.8)	0	2 (0.08)
Technical mistake in TKA	0	0	3 (0.1)
Other reasons	2 (1.5)	0	3 (0.1)
Total	32 (24)	0	23 (0.9)

Group 1: Patients prescribed antibiotics due to problems in the operated knee.

Group 2: Patients prescribed antibiotics for other reasons.

Group 3: Non-antibiotic group.

**Figure 2.**
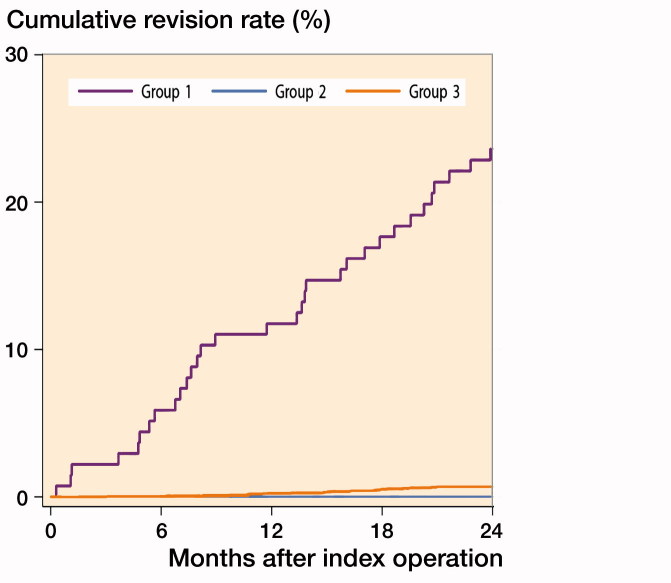
Cumulative revision rate of TKA due to infection in antibiotics users for reasons related to operated knee (Group 1), antibiotics users for other reasons (Group 2) and non-antibiotics group (Group 3).

The 2-year CRR after TKA in antibiotic users due to problems in the operated knee (Group 1) was 24% (95% CI 17–32) as compared with 0.7% (95% CI 0.4–1) among the no-antibiotics group (Group 3) (Figure 2).

## Discussion

Our results showed that 188 of the 2,769 TKA patients reported that they used antibiotics for more than 1 week, within 2 years after the primary procedure, and 132 of these antibiotics users reported that this was due to problems in operated knee. There are only a few reports in the literature investigating the success rate in curing periprosthetic infection using antibiotic therapy alone. Pavoni et al. (2004) used a non-operative approach to treat 34 patients with prosthetic joint infection (12 patients with early, 16 with delayed, and 6 with late infection). Most of the infections were initially treated with intravenous or intramuscular teicoplanin ± ciprofloxacin or rifampicin, followed by oral ciprofloxacin or minocycline plus rifampicin. 3 patients did not respond to therapy, and the infection was initially controlled in the remaining 31 patients. However, after longer follow-up (up to 5 years) less than half of the infected patients remained unrevised. In another study, Drancourt et al. (1997) reported a success rate of 52% for hips and 73% for knees when treating periprosthetic infection with a combination of antibiotics only, but the follow-up was short (up to 1 year after the therapy). Further, Drancourt et al. (1997) found that fusidic acid plus rifampicin cured 11 of 21 hip prosthesis infections and 8 of 11 knee prosthesis infections; in only 5 of 19 cured cases was removal of the device necessary. However, these studies are small, the success rate is not impressive, and they were performed before guidelines/consensus concerning the diagnosis and treatment of periprosthetic joint infections became commonly accepted.

There are no national guidelines regarding treatment of PJI in Lithuania; however, in the orthopedic departments dealing with PJI there is substantial knowledge on the topic, which is used as a basis for treatment decisions. According to the guidelines, the strategy in the treatment of PJI should be surgical intervention in combination with systemic antibiotics and not antibiotic treatment alone. These treatment pathways should be considered as a “gold standard” in the orthopedic community, but our study showed that this was not the case in Lithuania. Among the 132 TKA patients being treated with antibiotics because of problems with their knee, an orthopedic surgeon was the prescriber in 96 of the cases (74%).

Considering our finding that only 24% of the patients receiving antibiotics for more than 1 week became subject to revision within 2 years, it is probable that at least some of the patients did not have a true PJI because otherwise it is unlikely that 74% had escaped further surgery. Of 100 unrevised patients who received antibiotics for more than 1 week, only 9 had been subject to knee aspiration and cultures. That more than 1 week of antibiotic use must be considered treatment but not prophylaxis shows that antibiotics treatment was prescribed without relevant evaluation. The problem is that antibiotic therapy without proper diagnosis of a PJI, inclusive of cultures, not only reduces the possibility of choosing proper surgical and antibiotic treatment but also risks exposing patients to the wrong or unnecessary treatment and increasing bacterial resistance.

Of the 132 TKAs with signs of infection, only 22 had a subsequent revision for infection, while the other 110 remained unrevised. One can speculate that 110 knees might have avoided revision surgery. On the other hand, those not cured may have developed more severe infection, with resistant bacteria requiring more extensive surgery. Furthermore, widespread empiric treatment with broad-spectrum antibiotics will probably have a disadvantageous environmental impact, despite few patients being saved from revision surgery (Inabathula et al. 2018). Thus, targeted antibiotic therapy based on proper bacterial sampling is an essential part of appropriate treatment of PJI (Parvizi et al. 2011).

A drawback of our study is that we could not approach all the patients registered in the LAR. However, the proportion of interviewed patients was around 70% of the total number, which is why we assume that the results are a reasonable representation of the situation in Lithuania.

Another drawback is that the follow-up was only 2 years, as some patients with PJI who were treated with antibiotics only might still have low-grade infection with low symptom expression, thus being unrevised but not cured when the study ended.

We must bear in mind that it can be difficult to diagnose infection after TKA surgery, especially in non-hospital healthcare facilities. Thus, providing antibiotic treatment in the hope of the infection being “superficial” may be tempting, despite not being in accordance with widely accepted infection treatment protocols.

This is also supported by Wagenaar et al. (2017) who made a questionnaire-based online evaluation of current Dutch orthopedic care for persistent wound leakage after joint arthroplasty. Among 127 orthopedic surgeon respondents, 57% used a protocol for diagnosis and treatment of persistent wound leakage although only 27% utilized the protocol in every patient. However, 24% of orthopedic surgeons prescribed antibiotics due to wound problems. This suggests that improper use of antibiotics is not only a Lithuanian problem.

In summary, in Lithuania there seems to be a lack of adherence to evidence-based treatment guidelines when infection is suspected after primary TKA. By highlighting the problem and the spreading of information to both primary care and hospital staff the situation can be improved nationally and internationally.
